# Bioassay-Guided Fractionation of *Erythrostemon yucatanensis* (Greenm.) Gagnon & GP Lewis Components with Anti-hemagglutinin Binding Activity against Influenza A/H1N1 Virus

**DOI:** 10.3390/molecules27175494

**Published:** 2022-08-26

**Authors:** Tania Ortiz-López, Rocío Borges-Argáez, Guadalupe Ayora-Talavera, Ernesto Canto-Ramírez, Lisseth Cetina-Montejo, Ángel May-May, Fabiola Escalante-Erosa, Mirbella Cáceres-Farfán

**Affiliations:** 1 Unidad de Biotecnología, Centro de Investigación Científica de Yucatán, Chuburná de Hidalgo, Mérida 97205, Mexico; 2 Departamento de Virología, Centro de Investigaciones Regionales, Universidad Autónoma de Yucatán, Paseo de Las Fuentes, Mérida 97225, Mexico; 3 Independent Researchers, Mérida, Yucatán 97000, Mexico

**Keywords:** *Erythrostemon yucatanensis*, influenza A H1N1 virus, anti-hemagglutinin, sterols, flavone

## Abstract

*Erythrostemon yucatanensis* (Greenm.) Gagnon & GP Lewis is a legume tree native to and widely distributed in southeast Mexico, where its branches are used in traditional medicine. An in vitro evaluation of the antiviral activity of extracts and fractions from the leaves, stem bark and roots against two strains of the AH1N1 influenza virus was performed, leading to the identification of bioactive compounds in this medicinal plant. In a cytopathic effect reduction assay, the fractions from the leaves and stem bark were the active elements at the co-treatment level. These were further fractionated based on their hemagglutination inhibition activity. The analysis of spectroscopy data identified a combination of phytosterols (β-sitosterol, stigmasterol and campesterol) in the stem bark active fraction as the main anti-hemagglutinin binding components, while 5-hydroxy-2(2-hydroxy-3,4,5-trimethoxyphenyl)-7-metoxi-4H(chromen-4-ona), which was isolated from the leaf extracts, showed a weak inhibition of viral hemagglutinin. Time of addition experiments demonstrated that the mixture of sterols had a direct effect on viral particle infectivity at the co-treatment level (IC50 = 3.125 µg/mL). This effect was also observed in the virus plaque formation inhibition assay, where the mixture showed 90% inhibition in the first 20 min of co-treatment at the same concentration. Additionally, it was found using qRT-PCR that the NP copy number was reduced by 92.85% after 60 min of co-treatment. These results are the first report of components with anti-hemagglutinin binding activity in the genus *Erythrostemon* sp.

## 1. Introduction

Respiratory viral infections are a global health problem with a significant clinical impact. The high mutation capacity of viruses creates a race to find new and effective antivirals to combat novel viral pathogens. Medicinal plants are an essential source of chemical compounds with different biological properties. These natural sources of compounds have a myriad of applications in the pharmaceutical, food, perfume, agrochemical and cosmetics industries [1,2]. The World Health Organization classifies a medicinal plant as any that contains substances which can be used for therapeutic purposes, the whole or parts (e.g., roots, stem, leaves, stem bark, fruits and seeds) of which can have applications in the control or treatment of diseases, including respiratory infections [3].

The influenza virus presents a constant threat of a new pandemic: few antiviral options are available, and antiviral resistance even to the most novel influenza inhibitors has been detected [4]. Bio-directed studies using plant sources have mainly focused on the influenza virus’ surface viral proteins, neuraminidase (NA) and hemagglutinin (HA) [5,6] These glycoproteins are associated with virus transmission and pathogenicity [7]. HA recognizes sialic acid receptors in the respiratory mucosa, allowing virus adsorption into the cell. The sialidase activity of NA contributes to virus release from infected cells and virus dispersion within the host [8].

The *Erythrostemon* genus belongs to the *Caesalpinia* group, a large pantropical clade of about 205 plant species in the subfamily *Caesalpinioideae* (*Leguminosae*). Its genus delimitation has undergone considerable changes, and the current phylogeny-based classification of the *Caesalpinia* group recognizes 26 genera (*Erythrostemon, Cenostigma, Biancaea, Guilandina, Moullava, Caesalpinia*, etc.). Trees and shrubs in this group are used in traditional medicine as antimicrobials (*Cenostigma gaumeri* (Greenm.) E. Gagnon & G. P. Lewis) [9], antioxidants (*Biancaea sappan* (L.) Tod., Hort. Bot. Panorm) [10], analgesics and antihyperglycemics (*Guilandina bonduc* L.) [11], antidiabetics and antituberculars (*Moullava digyna* (Rottl.) E. Gagnon & G. P. Lewis) [12], anti-inflammatories (*Caesalpinia pulcherrima* (L.) Sw. [13] and for the treatment of different affections, including leprosy (*Caesalpinia crista* L. emend. Dandy & Exell) [14], the common cold and asthma (*Caesalpinia minax* Hance) [15]. Phenolic compounds with antiviral activity have been identified from *B. sappan*, including protossappanin A and B, brazilin, brazilein, 3-deoxysappanchalcone and sappanchalcone [11]. *Erythrostemon yucatanensis* (Greenm.) Gagnon & GP Lewis comprises 31 species, all widely distributed across the southern USA, Mexico and Central America [16,17]. In the Yucatan Peninsula in southeast Mexico, *E. yucatanensis* is widely used for fuel wood and its flowers are greatly appreciated for honey production [18]. In traditional Mayan medicine, it is used for the treatment of culturally related diseases. [19]. As part of a search for biologically active metabolites in the flora of southeast Mexico [20,21], the aim of this study was to evaluate the antiviral activity of extracts from *E. yucatanensis* throughout a bio-directed fractionation. The selection of active components was based on their ability to inhibit the virus’ hemagglutinin-binding activity.

## 2. Materials and Methods

### 2.1. Plant Material

**Figure 1:**
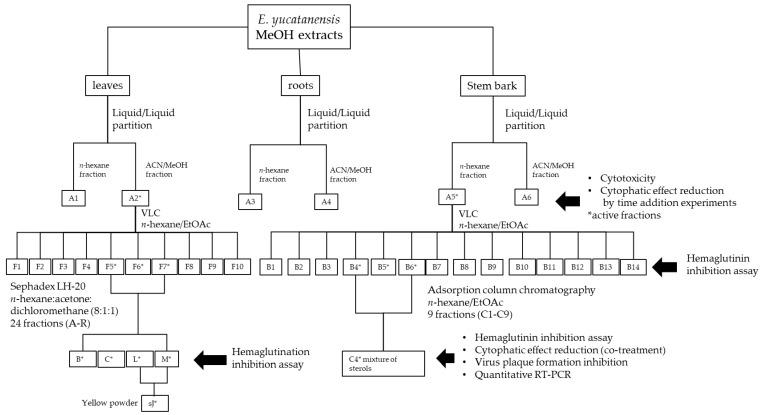
Experimental strategy for the bioassay-guided fractionation of antiviral compounds in *Erythrostemon yucatanensis*.

Plant material was collected in Yaxcabá municipality in the state of Yucatan, Mexico, by taxonomists from the Natural Resources Unit of the Scientific Research Centre of Yucatan (Centro de Investigacion Cientifica de Yucatán (CICY)). One specimen from each collection was archived in the Roger Orellana Herbarium (CICY) under the collection ID 2310. Stem bark, roots and leaves were dried under artificial light for 72 h at 60 °C, ground, weighed and stored in airtight bags. For the bioassay-guided purification (Figure 1), 3.06 kg of dried leaves, 1.02 kg of stem bark and 30 g of roots were extracted via static maceration in methanol (MeOH) for 24 h at room temperature. The extraction was filtered and concentrated under reduced pressure in a rotary evaporator at 40 °C.

### 2.2. Methanolic Extract Fractionation

The leaf (38.10 g), root (0.24 g) and stem bark (1.04 g) MeOH extract were processed with a liquid–liquid partition using MeOH/ACN/*n*-hexane, following the method described by Borges-Argáez et al. [22]. Briefly, each extract was dissolved in MeOH and ACN (acetonitrile), placed in a separation funnel and extracted with *n*-hexane at a 1:1:3 ratio (*v*/*v*/*v*); this was repeated three times. Both the *n*-hexane fraction and the ACN/MeOH fraction were obtained after drying under reduced pressure. Fractions A1 (*n*-hexanic extract), A2 (ACN/MeOH extracts) from the leaves, A3 (*n*-hexanic extract), A4 (ACN/MeOH extracts) from the roots, A5 (*n*-hexanic extract), and A6 (ACN/MeOH extracts) from the stem bark were selected for cytotoxic and antiviral evaluation (i.e., pre-treatment, co-treatment and post-treatment) (Figure 1).

### 2.3. Biological Assays

#### 2.3.1. Cells and Viruses

Madin–Darby canine kidney (MDCK) cells were kept in Dulbecco’s minimal essential media (DMEM) supplemented with 10% foetal bovine serum (FBS), 100 U/mL penicillin, 100 μg/mL streptomycin (GIBCO) and 1M HEPES and incubated at 37 °C in a 5% CO_2_ atmosphere. The influenza virus strains employed in the analyses were A/Yucatan/2370/09 (oseltamivir carboxylate susceptible) and A/MexicoInDRE/797/2010 (oseltamivir carboxylate resistant). They were cultured in MDCK cells, harvested at 72 h post-infection, aliquoted and stored at −80 °C until use.

#### 2.3.2. Cytotoxicity Assay

MDCK cells were grown in 96-well plates at a density of 1 × 10^5^ cells/well and incubated at 37 °C in 5% CO_2_ for 24 h. After washing the cells twice with phosphate buffer saline (PBS), 100 µL of each fraction (A1–A10) was placed in the wells at one of seven concentrations: 100, 50, 25, 12.5, 6.25, 3.125 and 1.56 µg/mL. Four replicates were carried out for each fraction at each concentration. As controls, each plate also had one well containing only DMEM. The plates were incubated for 72 h at 37 °C in 5% CO_2_. The inoculum was removed, and the cells were washed once with PBS, stained with 0.4% crystal violet in MeOH and incubated at room temperature for 30 min. After incubation, they were washed with running tap water and allowed to dry. Absorbance was measured at 490 nm using a Multilabel Plate Reader (Victor 3x Perkin-Elmer 2030, Perkim, Waltham, MA, USA). Cell viability was determined from the ratio between the optical density (OD) of the treated cells and the OD of the cell control considering 100% viable cells. %Cell viability = (*OD-treated cells/OD cell control*) × 100. The 50% cell cytotoxicity (i.e., the concentration causing cell death in 50% of the cells (CC_50_)) was calculated by plotting the extract concentration (µg/mL) versus the cell viability percentage and the curve regression analysis. Calculations were also performed to determine the maximum non-cytotoxic concentration (MCNC); that is, the maximum sample concentration with no cytotoxic effect and over 90% cell viability. Once the CC_50_ values for each fraction were calculated, a cytopathic effect reduction assay was performed.

#### 2.3.3. Cytopathic Effect Reduction Assay

Pre-treatment was run to determine if the extracts blocked viral infection. MDCK cells were grown in 96-well plates in DMEM supplemented with 10% FBS and incubated at 37 °C and 5% CO_2_ for 24 h. They were washed twice with PBS and incubated for another 24 h with 100 µL per well of the fraction (A1–A6) in quadruplicate. The inoculum was removed, and the cells were washed with PBS and incubated for 1 h at 37 °C in 5% CO_2_ with 50 µL of virus at 0.001 MOI for A/Yucatan/2370/2009 and 0.01 MOI for A/InDRE/797/2011. The virus was removed, and the cells were incubated for 72 h at 37 °C and 5% CO_2_ in 100 µL DMEM supplemented with 1 ug/mL TPCK trypsin.

Co-treatment was performed to evaluate if the fractions prevented the virus from binding to cell surface receptors. MDCK cells were grown in 96-well plates in DMEM supplemented with 10% FBS and incubated at 37 °C in 5% CO_2_ for 24 h. Different concentrations of the fractions A1–A6 were mixed with the respective viruses (0.001 MOI for A/Yucatan/2370/2009 and 0.01 MOI for A/InDRE/797/2011) and incubated for 1 h at room temperature. The virus + fraction mixtures (100 µL) were added to the cells and incubated for 1 h at 37 °C in 5% CO_2_, with four replicates per fraction. The inoculum was removed, DMEM supplemented with 1 µg/mL TPCK trypsin was added to each well, and the cells were incubated at 37 °C in 5% CO_2_ for 72 h.

Post-treatment evaluated if the extracts inhibited viral replication or blocked virus release from infected cells. MDCK cells were seeded in 96-well plates containing DMEM supplemented with 10% FBS and incubated at 37 °C in the presence of 5% CO_2_ until confluence. The cells were washed twice with PBS and infected with 100 uL of virus (0.001 MOI for A/Yucatan/2370/2009 and 0.01 MOI for A/InDRE/797/2011) and incubated for 1 h at 37 °C and 5% CO_2_. After the removal of the virus inoculum, the cells were incubated with 100 uL of different dilutions of the fractions (A1–A6) in DMEM supplemented with 1 ug/mL TPCK trypsin and incubated for 72 h in 5% CO_2_, with four well replicates per dilution. The cells were stained with 0.4% crystal violet in methanol for 30 min and washed with running tap water. Once dried, optical density (OD) was measured at 490 nm. In all the assays, the tested concentrations for fractions A1, A2, A3 and A6 ranged from 25 to 0.78 μg/mL, while for fractions A4 and A5, the concentrations ranged from 6.26 to 0.195 μg/mL. All assays were performed in triplicate. Oseltamivir carboxylate was used as a positive control. The percentage of viral inhibition was calculated with the following equation: viral inhibition = [(A−B)/(C−B)] × 100, where A is the OD of the infected cells, B is the OD of the virus control and C is the OD of the control cells. The mean inhibitory concentration of the viral effect (IC_50_) was calculated using linear regression.

#### 2.3.4. Hemagglutination Inhibition Assay

A hemagglutination inhibition (HI) assay was performed to evaluate if the components isolated from *E. yucatanensis* stem bark and leaves inhibited virus hemagglutination. The fractions were diluted in PBS at different concentrations (100, 50, 25, 12.5 and 6.25 µg/mL) by mixing 4 HAU of virus (in 25 µL) with the extract fractions (25 µL). These mixtures were placed in 96-well U-bottom plates and incubated for 1 h at room temperature. Soon after, 50 µL of 1% turkey erythrocytes solution was added to each well and incubated for 1 h. All assays were performed in duplicate. Controls were erythrocytes alone and a virus–erythrocyte mixture.

#### 2.3.5. Plaque Assay

MDCK cells were seeded in 12-well plates at a density of 1 × 10^6^ cells/well in DMEM supplemented with 10% SFB and incubated for 24 h at 37 °C in 5% CO_2_. The positive control was the influenza virus strain A/MexicoInDRE/797/2010 (MOI = 0.01) and synthetic glycosaccharide 6′SLN (6-sialyl-(N-acetylactosamine) [23] (Ayora-Talavera et al., 2014). Two mixtures, virus + C4 fraction (3.125 μg/mL) and virus + 6′SLN (157 μg/mL), were incubated at room temperature for six different time intervals (10, 20, 30, 40, 50 and 60 min). The mixture was added to the cells and incubated for 1 h at 37 °C to allow for virus adsorption. The mixture was removed, and the cells were incubated in 3% agar overlay media supplemented with 1 µg/mL TPCK trypsin at 37 °C and 5% CO_2_. After 72 h, the overlay media was removed, and the cells were stained with 0.4% crystal violet in MeOH for 30 min. Plaque formation inhibition was calculated as the plaque-forming units (PFUs) in each well compared to the virus control.

#### 2.3.6. RNA Extraction and Quantitative RT-PCR (qRT-PCR)

MDCK cells were grown in 12-well plates at a density of 1 × 10^6^ cells/well. The C4 fraction and 6′SLN were prepared at concentrations of 3.125 μg/mL and 250 μM, respectively, and co-treated with the influenza virus strain A/InDRE797/10 at MOI = 0. 01 for 10, 20, 30, 40, 50 and 60 min at room temperature before the cells were infected with the co-treated solutions at different times and incubated at 37 °C in a 5% CO_2_ atmosphere for 1 hr. Then, the inoculum was removed, 1 mL of DMEM supplemented with TPCK trypsin at a concentration of 1 μg/mL was added to each well and the cells were incubated for 24 hr at 37 °C in a 5% CO_2_ atmosphere. Subsequently, the cells and supernatant were harvested, and total viral RNA was extracted using the ROCHE Magna Pure Compact RNA Isolation kit (Roche, Basel, Switzerland) according to the manufacturer’s instructions. The RNA was stored at −80 °C until use. The detection and quantification of the NP gene was performed using real-time qRT-PCR with oligonucleotides (forward 5′GCA CGG TCA GCA CTT ATY CTR AG 3′; reverse 5′GTG RGC TGG GTT TTC ATT TGG TC3′) and a hydrolysis probe (TaqMan) (5′/56- FAM CYA CTG CAA GCC CA/BHQ) [24,25] with a quantitative qRT-PCR kit (One-Step Invitrogen SuperScript TM III Platinum One-Step). For the standard curve, the plasmid pDrive cloning vector QIAGEN containing the NP gene from strain A/InDRE797/10 was used as a control.

#### 2.3.7. Data Analysis and Interpretation

Data are presented as the mean ± standard deviation of three independent experiments. Statistical significance was calculated by a one-way ANOVA analysis and Dunnett’s test, with *p*-values < 0.05 considered as significant, using the GraphPad Prism 6.01 software.

### 2.4. Bioassay-Guided Fractionation of Leaf Extracts

The bioassay-guided fractionation was based on the hemagglutinin binding inhibitory activity of the extracts. The leaf extracts (38.10 g) were processed by liquid–liquid partition, which resulted in the A1 (11.60 g) and A2 (26.50 g) fractions, as previously described in Section 2.2. Fraction A2 was processed using VLC on silica gel (13 × 5 cm) eluted with 500 mL each of *n*-hexane (100%, 1 L, fraction 1–2) followed by 95:5 *n*-hexane:acetone (500 mL, fraction 3), 90:10 *n*-hexane:acetone (500 mL, fraction 4), 80:20 *n*-hexane:acetone (500 mL, fraction 5), 70:30 *n*-hexane:acetone (500 mL, fraction 6), 60:40 *n*-hexane:acetone (500 mL, fraction 7), 50:50 *n*-hexane:acetone (500 mL, fraction 8), 40:60 *n*-hexane:acetone (500 mL, fraction 9), 30:70 *n*-hexane:acetone (500 mL, fraction 10) and finally 100% acetone (250 mL, fraction 11). The resulting 15 fractions were compared using TLC and those with similar compositions were combined to obtain ten final fractions (F1–F10). Then, fractions F5, F6 and F7 (100 mg in total) were processed by gel permeation chromatography using a Sephadex LH20 (1.5 × 60 cm) and *n*-hexane:acetone:dichloromethane (8:1:1) as the mobile phase, resulting in 43 fractions. Based on the Rf value from the TLC results, 24 final fractions (A–R) were obtained. The fractions L and M yielded a yellow precipitate named sJ (5 mg), which was identified as a flavone using ^1^H-NMR and bidimensional analysis.

### 2.5. Bioassay-Guided Fractionation of Stem Bark Extracts

The bioassay-guided fractionation was based on the hemagglutinin binding inhibitory activity of the extracts. The partitioning of the stem bark extract (1.02 kg) produced the A5 (122.5 g) and A6 (925.2 g) fractions. A portion (40 g) of A5 was processed using VLC on silica gel (6 × 7 cm) and eluted with 500 mL of *n*-hexane (100%, 500 mL, fraction 1) followed by decreasing proportions of *n*-hexane:EtOAc (80:20, 70:30, 50:50, 25:75) until reaching 100% EtOAc. The column was washed with 100% MeOH (500 mL) to isolate 22 fractions. These were compared using TLC (*n*-hexane:EtOAc; 8:2 and 7:3) to produce 14 final fractions (B1–B14). Then, fraction B6 (1.03 g) was fractionated via adsorption column chromatography (3.5 × 30 cm) using silica gel (Sigma Aldrich, St. Louis, MI, USA) as the stationary phase, a 60 Å pore size and a 70–230 mesh. It was eluted with 250 mL each of *n*-hexane (100%, 250 mL) followed by decreasing proportions of *n*-hexane:EtOAc (100:00, 90:10, 80:20, 80:20, 75:25, 75:25, 50:50, 50) until reaching 100% EtOAc. The column was washed with 100% MeOH (250 mL), resulting in 261 fractions. These were compared using TLC (*n*-Hx: CH_2_Cl_2_:An; 8:1:1), which produced 9 final fractions (C1–C9) (Figure 1). A precipitate was isolated from fraction C4 and washed with MeOH, resulting in a white powder that was characterized using GC-MS.

### 2.6. Characterization of Anti-HA Compounds in C4

#### 2.6.1. Infrared Spectroscopy (FTIR-ATR)

The C4 fraction was also analysed via Fourier transform infrared spectroscopy (FTIR) using a spectroscope (BRUKER Tensor II, Madison WI, USA) with a diamond tip ATR detector. Data (Madison WI, USA) were collected within a 500–4000 cm^−1^ range with a resolution of 4 cm^−1^ and 32 scans in the specified wavelength range. The equipment automatically processed the data with an atmospheric CO_2_ compensation for each spectrum and an ATR diamond angle correction.

#### 2.6.2. Gas Chromatography-Mass Spectrometry (GC-MS)

The C4 fraction was analysed using GC-MS with a gas chromatographer (Agilent Technologies 7890A, Santa Clara, CA, USA) coupled to a mass detector (Agilent Technologies 5975C, Santa Clara, CA, USA) with a 5% phenyl- 95% methylpolysiloxane column (30 m long, 0.25 mm diameter, 0.25 µm film thickness) (Agilent Technologies 19091S–433HP-5MS, Santa Clara, CA, USA). Helium was the carrier gas, and the analysis parameters were as follows: initial temperature of 120 °C for 2 min, increase by 15 °C/min to 200 °C in 2 min, increase by 10 °C/min to 300 °C in 15 min and constant for 15+ min.

#### 2.6.3. NMR Structure Elucidation

^1^H, ^13^C, COSY, HMQC and HMBC NMR spectra were recorded with a spectrometer (Santa Clara, CA, USA) (Varian 600 MHz) in CDCl_3_ using TMS as an internal standard.

## 3. Results

### 3.1. Antiviral Activity of Leaf, Root and Stem Bark Fractions of E. yucatanensis

The antiviral activity of the leaf, root and stem bark fractions (A1–A6) was assessed in the different virus + extract treatments. The results of the cytotoxicity assay revealed that the leaf (A2) and stem bark (A5) fractions exhibited cytotoxic activity in the range of 41.70 to >100 μg/mL (Table 1). However, in the co-treatment (Table 1), the leaf and stem bark fractions were active against both virus strains with selectivity indexes >300, as indicated for A2. Thus, our results suggest that these fractions may affect the entrance of the virus into the cell via the interaction with viral hemagglutinin. The root fractions, A3 and A4, had the highest cytotoxicity. The cytopathic effect is the set of morphological changes or damage to the cells suffer being infected by a virus and that normally results in cell death. None of the fractions or extracts were active (IC50 > 6.25 μg/mL) in pre-treatment or post-treatment, although fraction A5 did exhibit some activity against the A/Mexico/Indre797/10 strain with an IC50 of 3.62 μg/mL. Therefore, the root extracts were excluded from the study and no further fractionation was performed.

### 3.2. Leaf Components from E. yucatanensis with Anti-HA Activity

**Figure 2:**
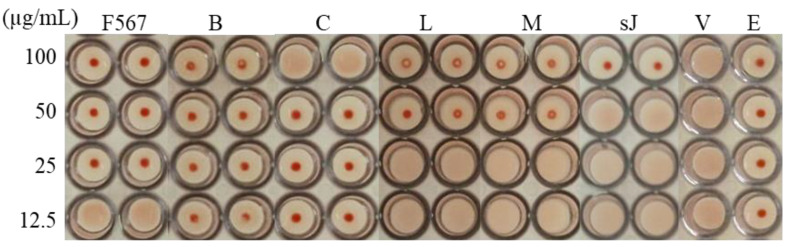
Hemagglutination inhibition activity of fractions F567, B, C, L and M and flavone sJ from the leaves of *E. yucatanensis*. Results: full inhibition (red button) or no inhibition (no red button). Controls: virus + erythrocytes (V) and erythrocytes (E).

The HI assay was used as an indirect approach to determine which leaf components showed anti-HA activity. From the bioassay fractionation, fractions F5, F6 and F7 exhibited the ability to inhibit hemagglutination at all tested concentrations (100, 50, 25 and 12.5 µg/mL) (Figure 2). These three fractions were combined (fraction F567), and the anti-HA activity was further evaluated in the 24 resulting fractions (A–R) (Figure 1). Fractions B, C, L and M were active, although only the yellow precipitate sJ, obtained from fractions L and M, showed weak inhibitory activity (Figure 2). The other fractions were not analysed further due to the small amount of sample obtained and their complexity, as determined by TLC (Figure 2). The pure sample was analysed using GC/MS, ^1^H NMR and bidimensional analysis. The ^1^H NMR analysis identified resonances for a strongly chelated OH group at δ12.55, typical of the 5-OH group; resonances for methoxyl groups between δ3.87 and 3.99 and aromatic protons; and a signal at δ7.34 typical of a flavone proton. A ^3^J_CH_ correlation between the methoxyl protons at δ3.81 and the carbon at δ165.59 was observed in the HMBC spectral data, which allowed the assignment of the methoxylated proton to the C−7 position. The HMBC data also identified a methoxyl at δ 3.99 attached to C4′ and a methoxyl at δ3.874 attached to C3′. The final methoxyl group at δ3.82 was assigned to the C5′ position. Additional OH group correlations were confirmed by the ^3^J_CH_ correlation between a δ12.55 proton and the carbons at δ97.79 (C6) and δ105.93(C4a) and by ^2^J_CH_ with δ161.84 (C5). The HSQC experiment identified eight protonated carbons. The substitution pattern on the A and B rings was fully established using NOE experiments with the irradiation of each methoxyl group. Based on this, the flavone sJ was unequivocally identified as 5-hydroxy-2(2-hydroxy-3,4,5-trimethoxyphenyl)-7-metoxi-4H(chromen-4-ona).

### 3.3. Stem Bark Fractions from E. yucatanensis with Anti-HA Activity

**Figure 3:**
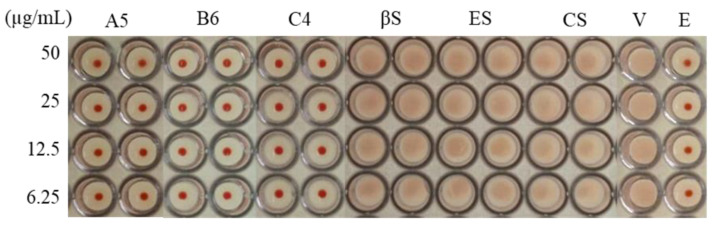
Hemagglutination inhibition assay of some stem bark fractions from *E. yucatanensis*. Results: full inhibition (red button) or no inhibition (no red button). HI assay of stem bark fractions (A5, B6 and C4) and analytical standards of β-sitosterol (βS), stigmasterol (ES) and campesterol (CS). Controls: virus + erythrocytes (V) and erythrocytes (E).

**Figure 4:**
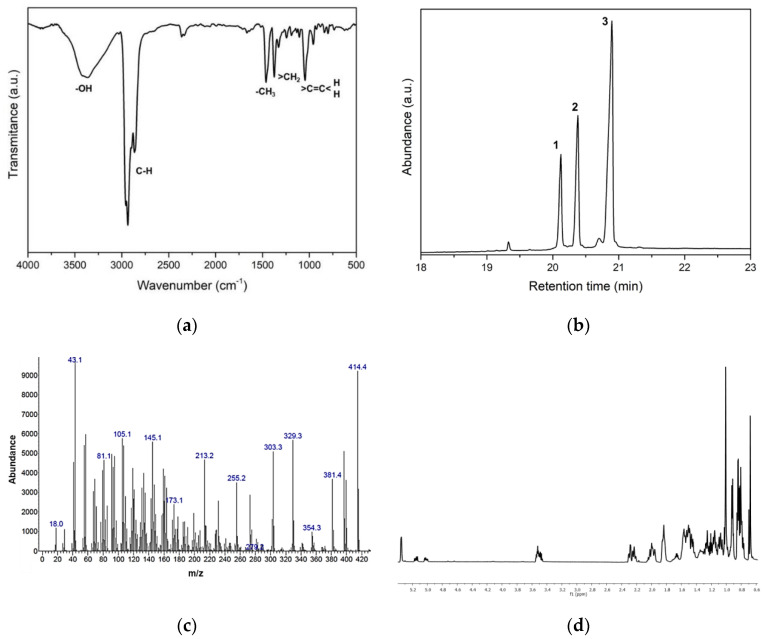
Characterization of anti-HA compounds in C4: (**a**) FTIR spectra of C4 showed absorption bands for hydroxyl groups at 3400 cm^−1^ as well as the tensile vibration of C–H sp3 bonds around 2900 cm^−1^. The bands found in the fingerprint region could be related to the vibrational modes of methyl, methylene and C=C double bonds. (**b**) GC-MS chromatogram of C4 showing three main peaks at retention times of 20.10 min, 20.50 min and 20.90 min that were characterized as campesterol (peak 1), stigmasterol (peak 2) and β-sitosterol (peak 3), with a similar molecular mass from *m*/*z* 414. (**c**) Chromatogram of the mass fragmentation (GC-MS) of C4, with a fragment at *m*/*z* 396, which corresponds to the OH group in position 3; an *m*/*z* 255 fragment, formed by the loss of the side chain attached to the sterane; and the fragments resulting from the loss of the side chain by the original sterane without water loss (273 *m*/*z*) and with the subsequent loss of 18 units due to the water molecule (OH group of position 3). (**d**) ^1^HNMR spectrum (CDCl_3_, 600 MHz) of the C4 fraction corresponding to a mixture of steroidal compounds.

The initial screening of the fractions from stem bark showed that A5 had some activity against the virus A/México/InDRE797/10. The bioguided fractionation resulted in 14 fractions (B1–B14), and fraction B6 also showed anti-HA activity in the HI assay at a concentration of 6.25 to 50 μg/mL (Figure 3). The sequential chromatographic fractionation of B6 ended with a final component named C4, which was a white crystalline powder that showed inhibition at a concentration of 6.25 µg/mL when evaluated in the HI assay. The C4 sample had a single spot on the TLC assay with an Rf of 0.31 corresponding to the 80:20 *n*-hexane:AcOEt elution system, suggesting that C4 was a pure compound. The IR analysis showed absorption bands for hydroxyl groups at 3400 cm^−1^ and the tensile vibration of C–H bonds around 2900 cm^−1^ (Figure 4a). However, the GC-MS analysis showed that C4 had three main peaks with RTs of 20.10 min (campesterol), 20.50 min (stigmasterol) and 20.90 min (β-sitosterol) (Figure 4b).

A typical fragment was observed in the mass fragmentation spectra at *m*/*z* 396, which corresponded to a fragment with a loss of 18 units due to a water molecule (from the OH group in position 3). This fragment underwent a new fragmentation, giving rise to the *m*/*z* 255 fragment, formed by the loss of the side chain attached to the sterane. The presence of these sterols was also confirmed by the fragments resulting from the loss of the side chain by the original sterane without water loss (273 *m*/*z*), and the subsequent loss of 18 units due to the water molecule (OH group in position 3) (Figure 4c).

The ^1^H NMR analysis revealed a profile that was characteristic of steroidal compounds. The series of intense high-field signals noted in the spectrum is associated with the presence of methyl groups at δ1.01 (s), δ 0.92 (d, *J* = 6. 4 Hz), δ 0.84 (d, *J* = 2.7 Hz), δ 0.83 (s), δ 0.82 (s), δ 0.81 (s) and δ 0.68 (s). The proton resonating at δ 3.51 (ddd, *J* = 19.2, 10.6, 6.8 Hz) corresponds to the proton of a carbon bonded to a hydroxyl group; this is a characteristic signal for the hydrogen located at position 3 of the sterane, which, according to the coupling constants, allows for the location of the hydroxyl group in the β-position. The signals at δ5.02 (dd, *J* = 15.1, 8.6 Hz, 3H) and δ5.15 (dd, *J* = 15.1, 8.7 Hz) are characteristic of the vinyl protons on the C-22 and C-23 carbons of stigmasterol, respectively. Another characteristic signal for sterols was present in the spectrum of a signal at δ5.35 (d, *J* = 2.8 Hz), which corresponds to the vinyl proton at position 6 of the sterane (Figure 4d). These data confirmed that the sample was a mixture of three compounds: campesterol, stigmasterol and β-sitosterol.

The pure standards of these sterols did not show inhibitory activity when evaluated in the HI assay (Figure 3) and at the co-treatment level in the cytopathic effect reduction assay (Table 2), in contrast to C4, which showed inhibitory activity in both assays with CI_50_ = 3.125 μg/mL and SI = 62.84. The cytotoxicity results showed that C4 and the sterols (β-sitosterol, stigmasterol and campesterol) had CC_50_ values higher than the maximum concentration measured (>100 μg/mL); thus, the SI was very low, suggesting that the activity identified in fraction C4 was likely a synergistic effect produced by the mixture of the three sterols identified.

### 3.4. Inhibition of Virus HA Binding and Infection by Component C4 from the Stem Bark of E. yucatanensis

**Figure 5:**
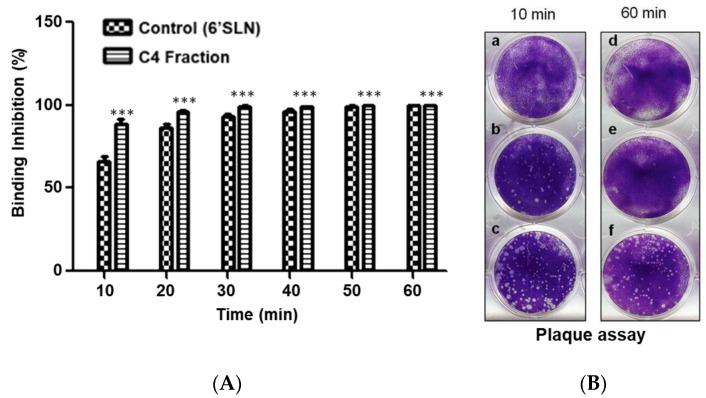
(**A**) Binding inhibition percentage of virus A/México/InDRE797/10 in MDCK cells at MOI = 0.01, co-treated at different times with the C4 fraction, *** *p*< 0.001. (**B**) Plaque assay of C4 fraction (a and d), 6′SLN (b and e) and viral control (c and f).

The inhibition of viral HA binding and MDCK cell infection was assessed using a plaque assay. In the presence of 6′SLN, plaque formation was inhibited at 60% after only a 10 min incubation (virus + 6′SLN) and reached 100% inhibition after 30 min. Interestingly, in the presence of 3.125 μg/mL of C4, this fraction’s inhibitory activity reached 90% at only 10 min of incubation and further increased to 100% after 30 min (Figure 5). The inhibitory effect of 6′SLN and the C4 fraction remained constant throughout the entire hour of incubation. Since plaque assays are used to quantify influenza virus replication rates, these results suggest that the components in the C4 fraction inhibited viral hemagglutinin binding to host cell sialic acid, thus preventing entry into the cell.

The effect of the C4 fraction on virus infection and replication was assessed by measuring NP gene copies in a standard curve using qRT-PCR. The results showed that in the presence of 6′SLN, the number of NP copies decreased in the first 10 min with 91.2% inhibition; after 20 min, NP synthesis was 100% inhibited with respect to the viral control. For the C4 sterol mixture, at 10 min of incubation the percentage of inhibition was 84.72%, while it reached 92.85% after 60 min of co-treatment (Table 3). These results suggest that C4 possesses an HA inhibitory activity that prevents binding to the cell receptor with a further effect on the infection and replication capacity of the H1N1 virus.

## 4. Discussion

The present study addressed the antiviral activity of *E. yucatanensis* extracts and pure compounds against the AH1N1pdm09 influenza virus and their possible mechanism of action. The influenza virus replication cycle initiates when viral HA binds to cell surface glycoconjugates containing terminal sialic acid residues [26]. Viral HA from human influenza strains preferentially recognizes receptors bound in an α−2,6 conformation (SAα2,6) to the sugar [27,28]. Our results revealed that the leaf and stem bark fractions were the most effective when added at the co-treatment level, having a significant effect on the inhibition of the binding capacity of viral HA on the MDCK cells’ surface.

The bioguided purification of the leaf fractions using an HI assay identified 5-hydroxy-2-(2-hydroxy-3,4,5-trimethoxyphenyl)-7-methoxy-4H(chromen-4-ona) as one of the components present in the active fractions. The results suggested that the activity of the isolated flavone increased when other components were present. Previous research has identified several methoxylated flavones with cytotoxic and anti-HIV-1 activities [29], first reported in leaves of *Gardenia carinata* from the *Rubiaceae* family [30]. There are no previous reports of this compound in members of the *Erythrostemon* group (reclassified into 26 genera); thus, this is the first report for a species *(E. yucatanensis)* in this group [16]. The isolation and identification reported in this study is a phytochemical contribution to the genera *Erytrosthemon.*

The plaque assay is one of the most reliable and commonly used methods to titre infectious virus particles. The effectiveness of a compound/extract/fraction in reducing the plaque size or plaque phenotype can be used as an indicator of its antiviral effect. The synthetic 6′SLN [23], used as positive control, inhibited virus plaque formation beginning at 10 min of incubation. At different co-treatment times, plaque formation was inhibited by the C4 fraction from *E. yucatanensis* stem bark as efficiently as observed in the presence of 6′SLN. It seems that blocking the virus from binding to the host cell affected viral infection, which was quantified by the number of NP copies, demonstrating that the inhibition of viral binding to the cell by the stem bark extract had a direct effect on viral replication. These results are relevant considering that there is a lack of drugs targeting viral binding to the host cell; these entry inhibitors might be considered as key targets for the development of new antivirals.

The bioguided purification of stem bark extracts helped us to characterize a mixture of sterols (β-sitosterol, stigmasterol and campesterol) as the constituents of the C4 fraction that effectively inhibited hemagglutinin in the HI assay and in the cytopathogenic effect reduction assay at the co-treatment level. An individual evaluation of the pure standards of these sterols did not show any antiviral activity, suggesting that the HA inhibitory activity resulted from a synergistic effect among these compounds. The identification of molecules of plant origin and, in particular, the synergistic effect of these molecules should be investigated deeply, as the greater efficiency of combinations of molecules with antiviral effects has been previously reported [31,32]. The most important plant sterols (commonly known as phytosterols) are stigmasterol, campesterol and β-sitosterol [33]. Due to their structural similarity, all three are widely used for various industrial and pharmaceutical applications, for example, as additives in functional foods, humectants, epithelial cell regenerators and cholesterol reducers. The anti-inflammatory effects of this phytosterol mixture have also been demonstrated in mice models. Their inhibition of pro-inflammatory cytokines is the main reason for their pharmacological effect [34].

Phytosterols similar to those reported in the present study have been isolated from extracts of the plants *Celastrus paniculatus Willd. (Celastraceae)* and *Aleurites moluccanus*. These extracts have been evaluated for their activity against the Newcastle disease virus (NDV) and avian influenza virus (AIV) H5N1, showing a 90% inhibition for the *A. moluccanus* extract in terms of viral titre reduction as evaluated by the HI assay [35]. However, only minimal research has been carried out on the activity of these compounds against influenza viruses [36].

## 5. Conclusions

The mixtures of leaf and stem bark extracts from *Erythrostemon yucatanensis* inhibited both the A/Yucatan/2370/09 (H1N1) and A/Mexico/InDRE797/10 (AH1N1) influenza virus strains, mainly by blocking viral entry into the host cells. Bioassay-guided fractionation based on influenza HI activity allowed for the identification of a mixture of β-sitosterol, stigmasterol and campesterol as the antiviral agent in the stem bark extract. This is the first report on the anti-hemagglutinin inhibition properties of bioactive compounds from *E. yucatanensis*. Natural source antivirals of this type are a valuable addition to the ongoing search for new antivirals.

## Figures and Tables

**Table 1:** Cytotoxic and antiviral activity of fractions A1 to A6 from *E. yucatanensis* during co-treatment assays.

Fractions	CC50	A/Yucatán/2370/09	A/México/InDRE797/10
(Plant part)	µg/mL	MOI:0.001	MOI:0.01
		IC50 (SI)	IC50(SI)
		µg/mL	µg/mL
A1 (leaves)	>100	>25 (4.00)	>25 (4.00)
A2 (leaves)	71.95	<0.195 (368.97)	<0.195 (368.97)
A3 (roots)	15.96	>6.25 (2.55)	>6.25 (2.55)
A4 (roots)	12.24	>6.25 (1.95)	>6.25 (1.95)
A5 (stem bark)	41.7	2.51(16.6)	4.83 (8.63)
A6 (stem bark)	50.47	>25 (8.04)	<0.78 (64.70)

CC_50_—50% cell toxicity concentration; IC_50_ 50%—inhibition concentration; SI—selective index, CC_50_/IC_50__._

**Table 2:** Cytotoxic and antiviral activity of C4, β-sitosterol, stigmasterol and campesterol.

Samples	CC50	A/Yucatán/2370/09	
	µg/mL	MOI:0.01	
		IC50	SI
		µg/mL	_CC50/IC50_
C4 (fraction)	196.36	3.125	62.84
β-sitosterol	>100	>100	<1
Stigmasterol	>100	>100	<1
Campesterol	>100	>100	<1

CC50—50% cell toxicity concentration; IC50—50% inhibition concentration; SI—selective index, CC50/IC50.

**Table 3:** Copy number of the NP gene of virus A/Yucatan/2370/09 co-treated with the C4 fraction or 6′SLN detected using qRT-PCR.

Time	Number of Copies		% Inhibition	
(min)	6′SLN	C4	6′SLN	C4
10	685.25	1189.51	91.2	84.72
20	0	1193.4	100	84.67
30	0	1112.74	100	85.7
40	0	1277.16	100	83.59
50	0	1334.84	100	82.85
60	0	556.51	100	92.85
* CV	7780.06			
* CC	0			

* CV—viral control. * CC—cell control.

## Data Availability

The data presented in this study are available on request from the corresponding author.

## Sample Availability

Samples of the compounds are not available from the authors.
